# Association of anthropometric indicators and blood pressure with subclinical cardiovascular diseases in obese adolescents: a cross-sectional study

**DOI:** 10.3389/fcvm.2025.1625575

**Published:** 2025-10-14

**Authors:** Xiaohui Liu, Shuang Xu, Jing Wang, Yan Feng, Yuwen Xia, Xiaofeng Yuan, Ruijing Tang, Kai Lu, Jun Shao

**Affiliations:** ^1^ Department of Ultrasound Diagnosis, Affiliated Kunshan Hospital of Jiangsu University, Kunshan, China; ^2^ School Health Department, Kunshan Municipal Centers for Disease Control and Prevention, Kunshan, China; ^3^ Disease Control and Prevention Division, Kunshan Municipal Health Commission, Kunshan, China; ^4^ Public Health Department, Affiliated Kunshan Hospital of Jiangsu University, Kunshan, China; ^5^ Department of Clinical Nutrition, Affiliated Kunshan Hospital of Jiangsu University, Kunshan, China

**Keywords:** carotid intima-media thickness, blood pressure, anthropometric indicators, risk factors, cross-sectional study

## Abstract

**Background:**

Carotid–femoral pulse wave velocity, carotid intima–media thickness (cIMT) and left ventricular hypertrophy are early measures of future subclinical cardiovascular disease (CVD) events. Although the associations between body mass index (BMI), abdominal adiposity measures, blood pressure, and CVD have been relatively well studied in adults, the evidence in children and adolescents is limited. Therefore, this study aimed to investigate the associations between BMI, abdominal obesity indices, blood pressure levels, and cIMT among obese adolescents in Jiangsu Province, China, and to identify potential critical thresholds.

**Methods:**

A stratified cluster sampling method was used to select participants from primary, middle, and high schools in Jiangsu Province. Clinical examinations included anthropometric measurements, blood sampling, and ultrasound assessments. cIMT was measured via carotid ultrasound. Statistical analyses, including Spearman correlation coefficients, nonlinear fitting, piecewise regression and multiple linear regression, were conducted to explore the associations between BMI, abdominal obesity indicators, blood pressure levels, and cIMT in obese adolescents.

**Results:**

Among the 245 obese adolescents, the waist‒to-hip ratio (WHR), BMI, systolic blood pressure (SBP), and diastolic blood pressure (DBP) were significantly positively correlated with cIMT (*P* < 0.001).Nonlinear fitting and piecewise regression revealed that right cIMT increased sharply with SBP > 115.7 mmHg, DBP > 70.9 mmHg, BMI > 26.0 kg/m^2^; left cIMT increased sharply with SBP > 131.0 mmHg, DBP > 81.8 mmHg, BMI > 33.0 kg/m^2^, and WHR > 0.84. Multivariate analysis indicated that only SBP maintained an independent association with left cIMT (*P* < 0.05).

**Conclusion:**

Our study revealed a significant association between anthropometric measures and cIMT in obese adolescents, and these factors can be used as early markers of subclinical CVD. Early intervention for BP control may help reduce long-term CVD risk in this population.

## Introduction

The carotid-femoral pulse wave velocity, carotid intima-media thickness (cIMT), and left ventricular mass index are well-established surrogate markers for subclinical cardiovascular disease (CVD), primarily coronary heart disease and stroke (1). In adult populations, the associations between body mass index (BMI), abdominal obesity indicators, blood pressure levels, and CVD incidence have been extensively documented (2, 3). However, extrapolating these findings to younger populations is problematic due to distinct physiological and developmental trajectories (4).

Despite the rising global prevalence of childhood obesity, a critical public health concern, research examining its direct impact on early vascular aging in adolescents remains limited, both domestically and internationally (5–7). This gap is particularly salient in China, where rapid socioeconomic changes have accelerated obesogenic environments (8). While numerous studies have focused on adult CVD risk factors, comprehensive data on the precise relationships between anthropometric measures, blood pressure, and cIMT in obese adolescents are scarce (9, 10).

As one of China's most economically developed and populous provinces, Jiangsu Province represents an ideal and strategic location for this investigation. This economic prosperity, however, is associated with lifestyle changes that may increase the risk of childhood obesity (11). No prior study has specifically explored the relationship between anthropometric indicators, blood pressure, and subclinical CVD in obese adolescents within this populous and economically significant region.

Research has revealed a significant correlation between BMI, abdominal obesity indicators, and blood pressure levels during childhood and adulthood (12–14). Therefore, elucidating the associations among BMI, abdominal obesity indicators, and blood pressure levels with cIMT in adolescents is biologically plausible and critical for early intervention. Furthermore, as CVD is a major chronic disease affecting the health of the Chinese population, identifying early biomarkers and risk thresholds during childhood has substantial practical and public health value (13, 15).Based on this background and rationale, this study was specifically designed to (1) investigate the associations between BMI, abdominal obesity indicators, blood pressure levels, and cIMT in obese adolescents; (2) identify potential critical thresholds for these metrics beyond which the risk of increased cIMT rises significantly; and (3) evaluate which risk factors maintain an independent association with subclinical atherosclerosis after adjusting for potential confounders.

## Methods

### Study population

Our study selected participants from primary, middle and high schools in Jiangsu Province (located between 116°21′E - 121°56′E and 30°45'N - 35°08′N) via stratified cluster sampling, thus ensuring representation across gender and age groups. Weight status was assessed via age- and sex-specific BMI percentiles, and obese children (defined as BMI ≥ 95% for age and sex) were included in further analyses (16).The sample size was determined through a statistical power calculation using the formula for estimating proportions in a population, which is a standard method in clinical and epidemiological research. This formula ensures the sample is large enough to detect a statistically significant effect with a high degree of confidence, while remaining feasible to recruit. The formula used was: $n\;=\;\frac{Z^{2}\cdot p(1-p)}{E^{2}}$, Where: *n* = required sample size, Z = *Z*-score corresponding to the desired confidence level. We set this at 95% (a standard in medical research), corresponding to a *Z*-score of 1.96. *p* = estimated proportion (prevalence) of the key characteristic in the population (17). Baseline data from the study population were collected through standardized clinical examinations, including anthropometry, blood sampling, and ultrasound evaluation. All anthropometric measurements and clinical indicators were objectively obtained during these physical examinations conducted in a hospital setting, using calibrated instruments and following strict protocols. Ethical approval for this study was obtained from the institutional review boards of Kunshan Municipal Centers for Disease Control and Prevention (approval number: 2024020), and informed consent was obtained from the participants and their parents or guardians.

### Measurement

We measured several key indicators, including exposure factors such as height, body mass, waist circumference, hip circumference, systolic blood pressure, and diastolic blood pressure, as well as the following outcome factors: cIMT. Height and body mass were measured via a calibrated digital scale, with each child wearing light clothing and without shoes or hats. BMI was calculated as body mass (kg) divided by the square of height (m^2^). Waist circumference was measured at the midpoint between the lowest point of the costal arch and the upper edge of the iliac crest, using a flexible tape measure horizontally around the body, ensuring that the tape was level and untwisted; the measurement was recorded where the tape intersected with the 0 mark. Hip circumference was measured at the most prominent point of the gluteus maximus at the front and back, with the tape snugly enciring the measurement point without compressing the skin, and the measurement was recorded accordingly. The waist‒to-hip ratio (WHR) was calculated by dividing waist circumference (cm) by hip circumference (cm).

Blood pressure measurements were conducted via an automatic electronic blood pressure monitor, which inflates and deflates automatically. Once the measurement was completed, the monitor displayed the systolic blood pressure (SBP), diastolic blood pressure (DBP), and pulse rate on the screen. To ensure accuracy, we took two measurements, each separated by an interval of 30–60 s. If the difference in SBP or DBP readings between these two measurements exceeded 5 mmHg, we performed a third measurement and recorded the average of all three readings.

cIMT was assessed via carotid ultrasound. Grayscale features of the outer membrane region were extracted from the collected carotid ultrasound images, and the intima–media boundary and lumen–intima boundary lines were delineated to calculate the cIMT on the basis of these boundaries. The average cIMT was calculated from three measurements taken from both the left and right carotid arteries.

### Statistical analysis

In our study, the distribution of all continuous variables was first assessed using the Shapiro–Wilk test. Variables that were normally distributed are presented as mean ± standard deviation (SD), while non-normally distributed variables are presented as median (interquartile range, IQR). This approach was applied to understand the baseline characteristics of the population by age stratification (≤12 years, prepubertal; >12 years, postpubertal). The distribution of cIMT across age and sex was analyzed via scatter plots and linear fitting. Bivariate associations between anthropometric parameters and cIMT were assessed via Spearman's rank correlation coefficient. For comparisons of continuous variables that were not normally distributed across three or more groups, the Kruskal–Wallis test was employed. The locally weighted scatterplot smoothing (LOESS) method was used to explore the nonlinear relationships between variables (18), which were visualized via scatter plots and smooth curves. To precisely identify the turning points in the relationships between cardiometabolic indicators (SBP, DBP, BMI, WHR) and cIMT, we employed piecewise regression analysis using the segmented package in R (19). This method iteratively fits two linear regression models to the data on either side of a potential break point and estimates the location of the breakpoint that minimizes the residual sum of squares for the entire model. Multiple linear regression analysis was used to determine independent risk factors affecting cIMT: Model 1 did not adjust for any variables, Model 2 adjusted for core demographic characteristics (age, sex), and Model 3 adjusted for all covariates except the measure itself. To control the Type I error (false positive result) in multiple hypothesis testing, the significance level (α) was set at 0.05 and adjusted using the Bonferroni correction where appropriate for multiple comparisons. All analyses were conducted in R 4.4.1. Statistical significance was defined as a two-tailed *P*-value < 0.05.

## Results

### Characteristics of the study population

According to the formula for calculating the sample size, p was the prevalence of obesity in our study population. Based on national data indicating a childhood obesity prevalence of approximately 17%–20% (8, 20) (17.06% according to statistics from Kunshan, Jiangsu Province, Supplementary Material 1), we used *p* = 0.20 (20%) for a conservative estimate. E = desired margin of error (precision). We set this at ±5% (0.05), meaning we can be 95% confident that the true population parameter lies within 5% of our sample estimate. Calculation: $\;n=\frac{1.96^{2}\cdot 0.2(1-0.2)}{0.05^{2}}=245.86$. A total of 245 obese adolescents with a mean age of 12 years were included in this study. Most participants were boys (*n* = 172, 70.2%), with a mean BMI of 25.5 kg/m^2^, a median SBP of 118.67 mmHg, a median DBP of 68.67 mmHg, and a median WHR of 0.83. Table 1 details the anthropometric characteristics of obese adolescents by sex and age group. The results of the Kruskal‒Wallis test revealed significant differences in related indicators between different sex and age groups.

**Table 1:** Baseline characteristics of obese adolescents.

Type	Gender	Age ≤12 (years) (*N* = 131)		Age >12 (years) (*N* = 114)		Kruskal‒Wallis *P*
		Median/mean	IQR/sd	Median/mean	IQR/sd	
Systolic blood pressure (mmHg)	F	105.50	12.08	124.33	14.33	<2.2E-16
	M	111.66	13.83	132.67	17.00	
Diastolic blood pressure (mmHg)	F	60.33	13.08	73.33	7.33	2.7E-16
	M	64.00	8.00	73.67	12.33	
Waist hip ratio	F	0.76	0.06	0.88	0.09	<2.2E-16
	M	0.79	0.07	0.89	0.08	
Body mass index (kg/m^2^)	F	21.73	3.44	28.79	4.7	<2.2E-16
	M	23.31	3.78	28.70	4.51	

**Figure 1:**
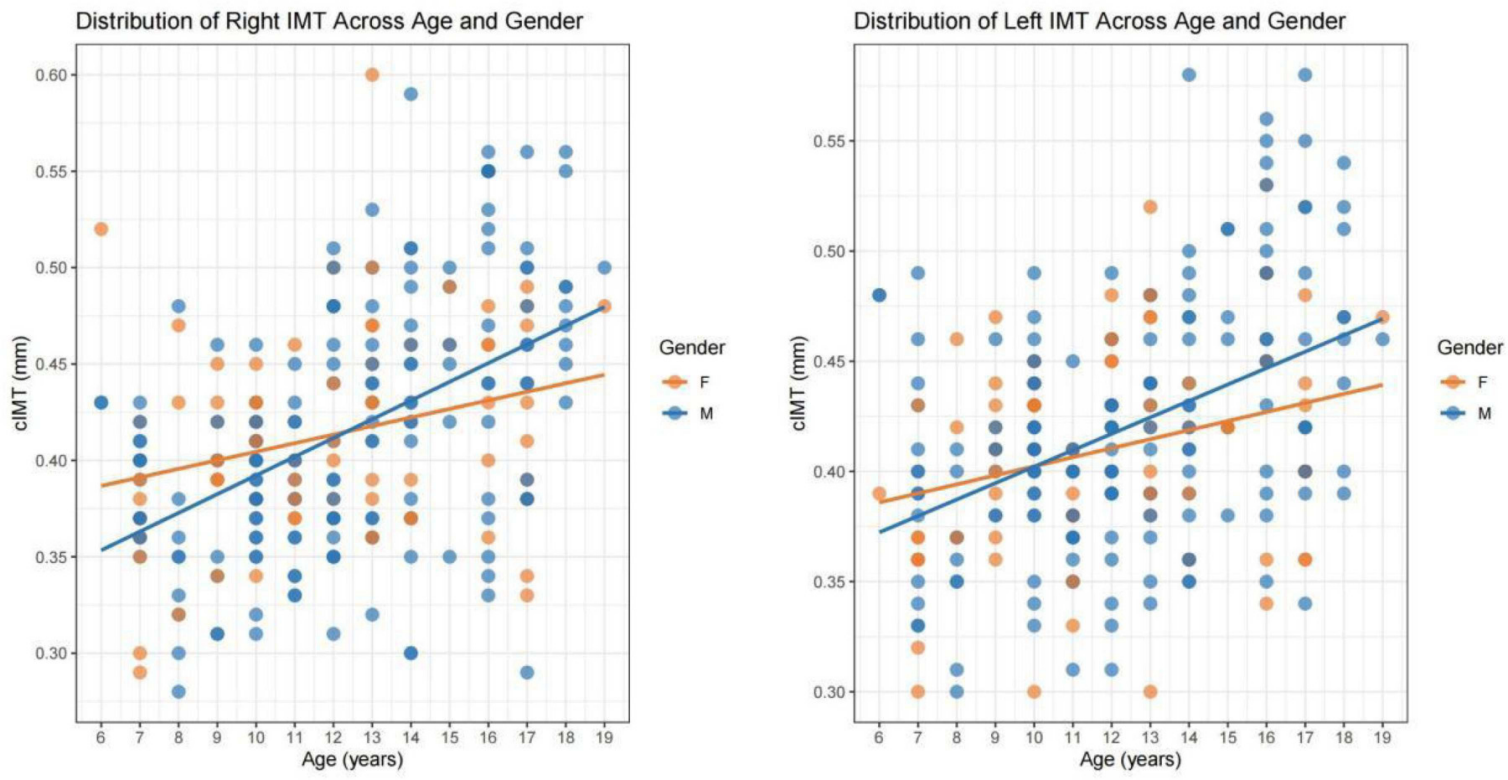
Distribution of cIMT across age and gender.

Figure 1 shows the distribution of cIMT in obese children by age and sex. The mean left IMT was 0.42 mm and ranged from 0.30 to 0.58 mm, whereas the mean right IMT was 0.41 mm and ranged from 0.28 to 0.60 mm. Notably, after the 10‒12 years of age, males presented higher cIMT values than females did, suggesting a possible sex difference in carotid atherosclerosis progression.

### Correlation analysis

Correlation analysis revealed that the waist‒hip ratio, BMI, systolic blood pressure and diastolic blood pressure were positively correlated with cIMT (*P* < 0.001). The Spearman correlation coefficients are shown in Table 2. The results revealed that increased anthropometric indices were strongly associated with elevated cIMT, suggesting their potential role in the development of early vascular changes.

**Table 2:** Spearman correlation coefficients between anthropometric indicators, blood pressure, and cIMT.

Correlation	Left IMT		Right IMT	
	Coefficients	*P*	Coefficients	*P*
WHR	0.425	5.74E-12	0.447	2.92E-13
BMI	0.446	3.31E-13	0.455	9.72E-14
SBP	0.417	1.49E-11	0.418	1.25E-11
DBP	0.306	1.27E-06	0.286	6.65E-06

### Nonlinear fitting and piecewise regression analysis

**Figure 2:**
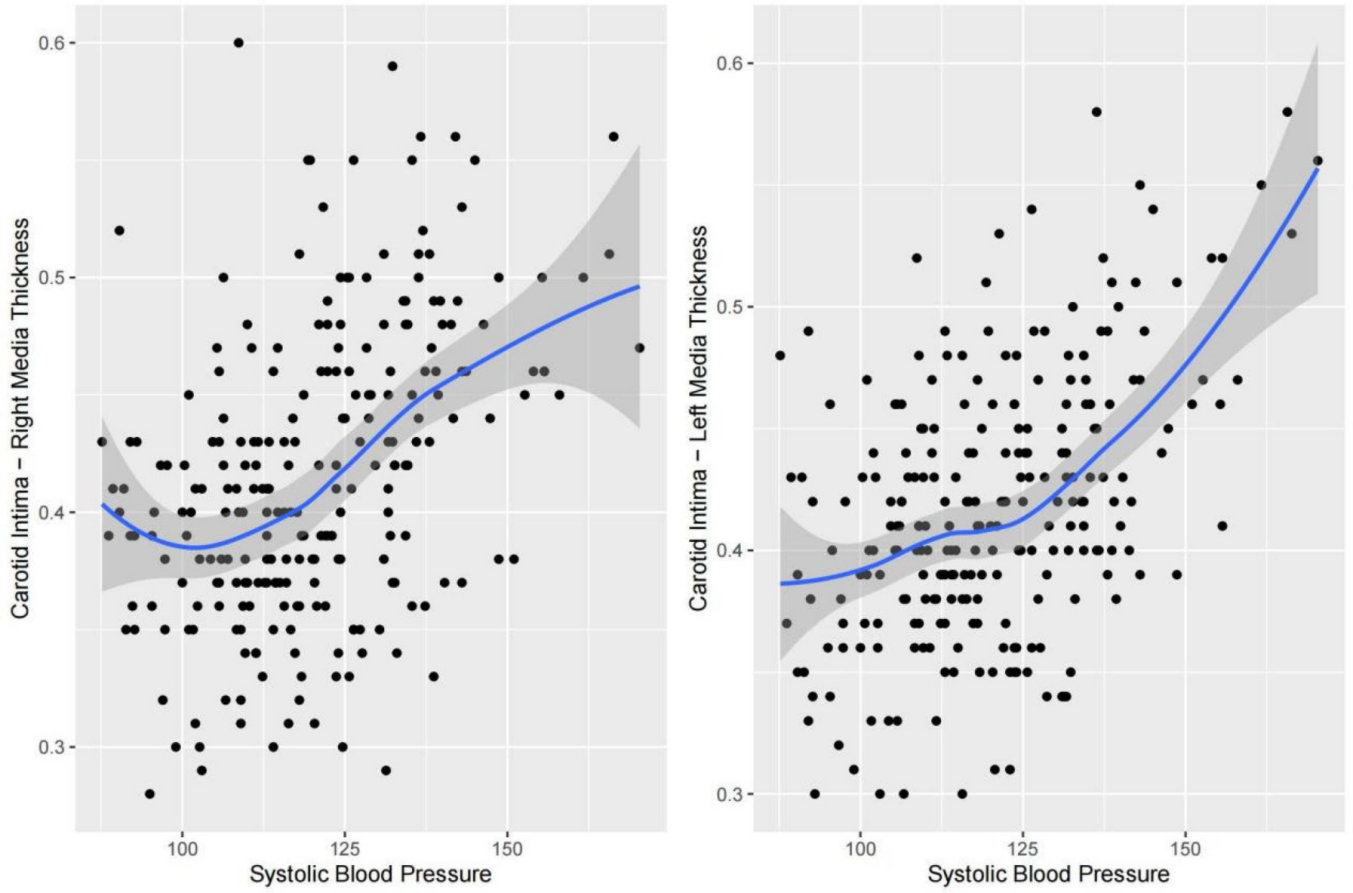
Dose‒response relationships between systolic blood pressure and cIMT.

**Figure 3:**
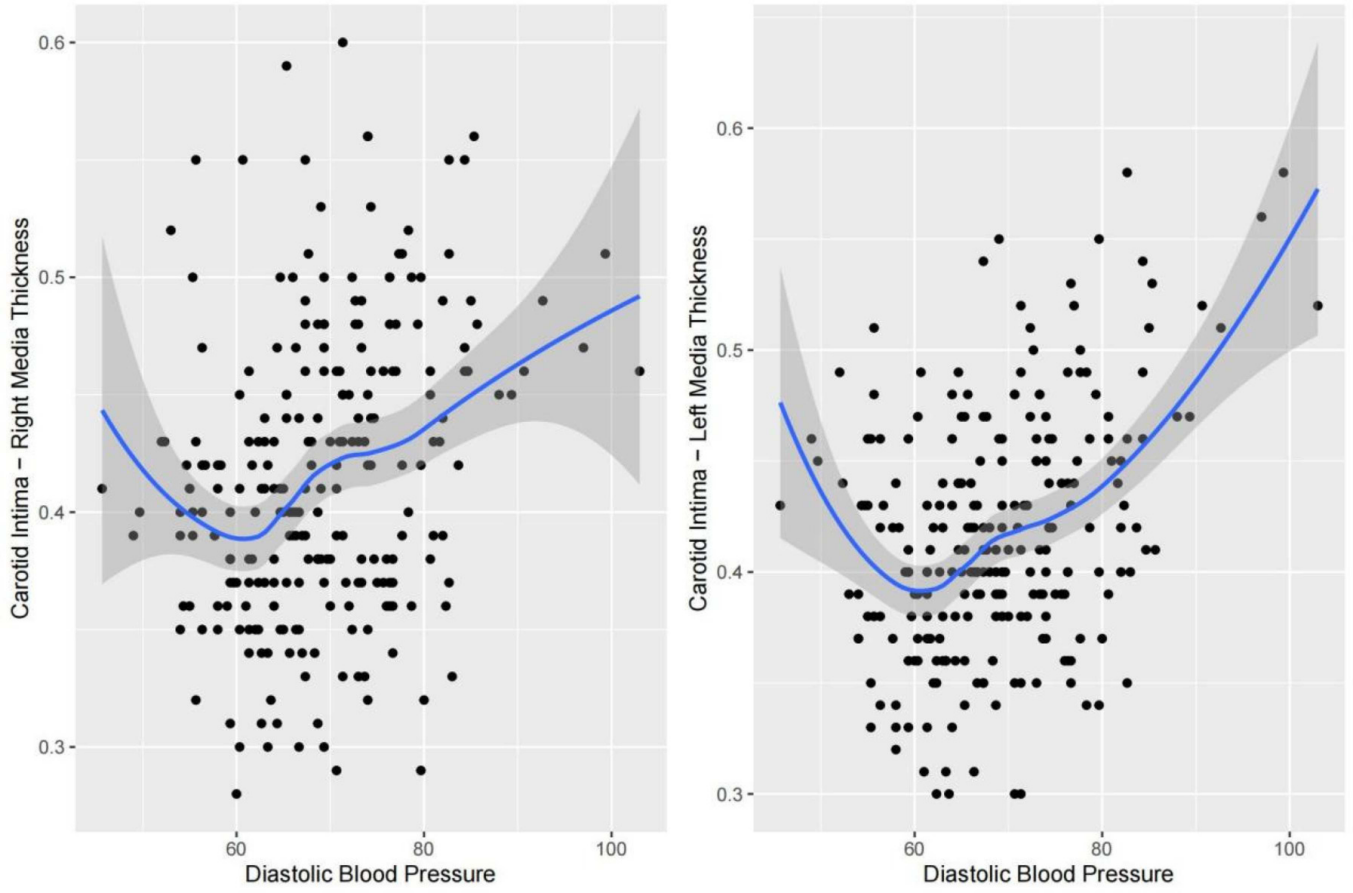
Dose‒response relationships between diastolic blood pressure and cIMT.

**Figure 4:**
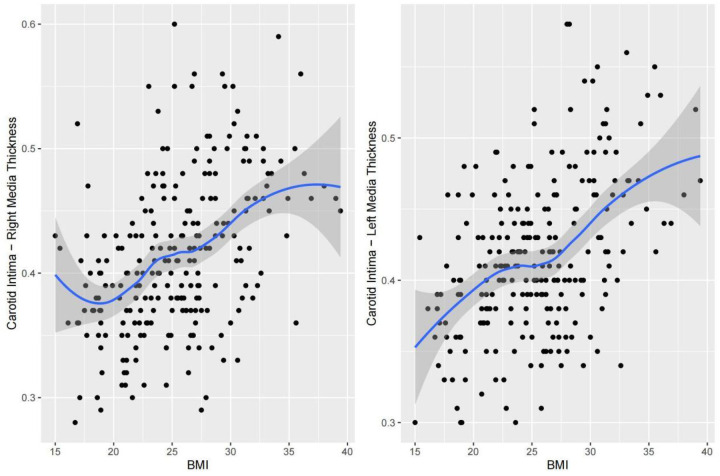
Dose‒response relationship between BMI and cIMT.

**Figure 5:**
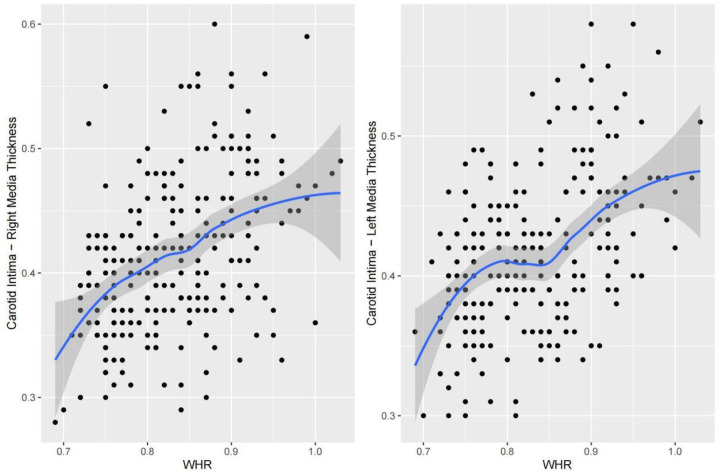
Dose‒response relationship between the waist‒to-hip ratio and cIMT.

Nonlinear fitting was performed to analyze the trends between each indicator and cIMT. The piecewise regression analysis identifies exact inflection points under different trends. For SBP, right cIMT remained relatively stable between 95.0 and 115.7 mmHg but showed a steep increase as SBP exceeded 115.7 mmHg; left cIMT remained relatively stable between 93.3 and 131.0 mmHg but showed a steep increase as SBP exceeded 131.0 mmHg (Figure 2). With respect to DBP, a multi-phase association was also observed. Right cIMT decreased as DBP increased from 40.0 to 60.0 mmHg, and then increased markedly above 70.9 mmHg. Left cIMT declined with increasing DBP up to 58.7 mmHg, and increased rapidly above 81.8 mmHg (Figure 3). In terms of BMI, the association with cIMT was complex and side-dependent. Right cIMT decreased gradually until BMI reached 22.3 kg/m^2^, and then rose above 26.0 kg/m^2^. Left cIMT showed a different trajectory, with a increase until 21.0 kg/m^2^, remained relatively stable between 21.0 and 23.0 kg/m^2^, and a sharp rise above 23.0 kg/m^2^ (Figure 4). The relationship between WHR and cIMT also demonstrated lateral differences. Right cIMT remained steadily rising during the increase in WHR. Left cIMT showed a stable phase between WHR 0.78 and 0.84, and a rapid increase above 0.84 (Figure 5).

### Multivariate analysis

To comprehensively evaluate the independent effects of each index on cIMT, multivariate logistic regression analysis was performed. The analysis revealed that, after adjusting for other covariates, SBP was the only anthropometric measure among those analyzed that maintained a statistically significant independent association with cIMT (*P* < 0.05), as presented in Tables 3, 4. This finding suggests that SBP may be a key target for interventions to reduce cardiovascular risk in this population.

**Table 3:** Multivariate analysis of anthropometric indicators and blood pressure in the Right IMT.

Right IMT	Unadjusted				Adjusted age and gender				Adjusted other vars			
	Estimate	SE	*t*	*P*	Estimate	SE	*t*	*P*	Estimate	SE	*t*	*P*
WHR	0.377	0.048	7.727	2.9E-13	0.241	0.069	3.458	6E-4	0.132	0.102	1.288	0.198
BMI	0.005	0.001	7.695	3.5E-13	0.003	0.001	3.382	8E-4	0.001	0.002	0.882	0.378
SBP	0.002	0.000	7.297	4.2E-12	0.001	0.000	2.883	0.004	0.001	0.000	1.936	0.053
DBP	0.002	0.000	4.582	7.4E-6	0.000	0.000	0.673	0.501	−0.001	0.001	−1.496	0.135

**Table 4:** Multivariate analysis of anthropometric indicators and blood pressure in the left IMT.

Left IMT	Unadjusted				Adjusted age and gender				Adjusted other vars			
	Estimate	SE	*t*	*P*	Estimate	SE	*t*	*P*	Estimate	SE	*t*	*P*
WHR	0.336	0.043	7.833	1.5E-13	0.262	0.062	4.218	3.5E-5	0.063	0.090	0.706	0.480
BMI	0.005	0.001	8.321	6.4E-15	0.004	0.001	4.777	3.1E-6	0.002	0.001	1.761	0.079
SBP	0.002	0.000	8.338	5.7E-15	0.001	0.000	4.686	4.6E-6	0.001	0.000	2.181	0.030
DBP	0.002	0.000	6.159	3.0E-9	0.001	0.000	2.901	4.1E-3	0.000	0.001	0.043	0.905

## Discussion

Our study revealed a significant association between anthropometric measurements and cIMT in obese adolescents and further explored the nonlinear relationships among the WHR, BMI, blood pressure and cIMT, as well as their independent effects on cIMT. Given the increasing prevalence of obesity and hypertension among children and adolescents globally (21, 22), early screening and intervention strategies are urgently needed to reduce cardiovascular risk in the future.

The observed ratio of approximately 70% boys to 30% girls in our cohort of 245 obese children is not a result of sampling bias, but rather an accurate reflection of the underlying epidemiological reality of childhood obesity in our study region. Our sample's gender distribution aligns almost perfectly with the obesity prevalence data from our region (Supplementary Material 1). The provided data indicates: Boys' obesity prevalence = 21.19%, Girls' obesity prevalence = 12.33%. Our observed ratio of ∼70% is slightly higher than this theoretical expectation but falls well within a reasonable range of sampling variation, especially when considering that childhood obesity rates are consistently reported to be higher in boys than girls (11, 12). This confirms that our sample's composition is a representative and accurate reflection of the local childhood obesity landscape. Interestingly, boys entering the pubertal stage had higher cIMT values than girls of the same age did (Figure 1). These findings imply that sex and age may play a role in the progression of carotid atherosclerosis. This finding is consistent with previous studies showing that males are more prone to vascular changes after puberty, the reason behind this may be the effect of sex hormones on vascular remodeling around puberty, as well as differences in fat distribution patterns and changes in lifestyle factors (23–25).

Some studies have noted that subclinical damage in different parts of the arterial tree is not uniformly affected by the same cardiovascular risk factors (26, 27). Therefore, we measured the bilateral carotid arteries and evaluated the influence of anthropometric indicators. However, our study revealed that WHR, BMI, and blood pressure were positively correlated with bilateral cIMT. Similar results have been reported in studies of children and adults (9, 28–30). Nonlinear fitting and piecewise regression revealed several turning points, those were associated with elevated cIMT. These cutoff points are consistent with the stage II diagnostic criteria in the latest ACC/AHA adolescent hypertension guidelines and the diagnostic threshold for abdominal obesity recommended by the WHO (29, 31–36), However, it is noteworthy that our piecewise regression analysis revealed even more nuanced, side-specific turning points, suggesting that the relationship between risk factors and vascular changes may be more complex than a single universal threshold. Multivariate analysis further highlighted the critical role of SBP as an independent predictor of cIMT. Although other factors, such as BMI and the WHR, were strongly associated with cIMT, only systolic blood pressure maintained an independent relationship with cIMT after accounting for potential confounders. This finding highlights the critical importance of blood pressure management as a primary intervention strategy (37, 38).

Our study boasts several strengths that enhance the validity and impact of our findings. Firstly, the use of a stratified cluster sampling method improved the representativeness of our sample of obese adolescents. Secondly, all data were derived from objective and precise clinical measurements (e.g., calibrated scales, ultrasound for cIMT), eliminating the potential for recall bias inherent in self-reported data and ensuring high data accuracy. Thirdly, employing advanced statistical techniques—specifically, nonlinear fitting and piecewise regression analysis—allowed us to identify precise risk thresholds beyond which cIMT increased markedly. Lastly, by focusing on the critical yet understudied population of obese adolescents, our research provides valuable insights for crafting targeted early prevention strategies, addressing a significant public health gap. Collectively, these strengths underscore the robustness of our study design and the credibility of our conclusions.

However, our study has several limitations. First, this was a cross-sectional study, and no conclusions can be drawn about causality. Future studies should focus on longitudinal studies to track the progression of cIMT and other subclinical CVD markers over time. In addition, it is necessary to incorporate other factors in addition to traditional cardiovascular risk factors, such as physical exercise and genetic factors, to study the impact of these indicators on CVD risk.

In conclusion, our study establishes significant nonlinear relationships between obesity-related indicators (BMI, WHR) and blood pressure (SBP, DBP) with cIMT in adolescents, identifying critical thresholds that trigger accelerated vascular damage. Systolic blood pressure independently predicts cIMT progression, highlighting its pivotal role in early cardiovascular risk assessment. These findings advocate for prioritized blood pressure management and obesity interventions in youth, supported by public health strategies promoting lifestyle modifications.

## Data Availability

The raw data supporting the conclusions of this article will be made available by the authors, without undue reservation.
